# Platelet factor 4 (CXCL4/PF4) upregulates matrix metalloproteinase-2 (MMP-2) in gingival fibroblasts

**DOI:** 10.1038/s41598-022-19850-w

**Published:** 2022-11-03

**Authors:** Hoa T. Le, Kalyan Golla, Ryan Karimi, Michael R. Hughes, Flavia Lakschevitz, Douglas B. Cines, M. Anna Kowalska, Mortimer Poncz, Kelly M. McNagny, Lari Häkkinen, Hugh Kim

**Affiliations:** 1 Department of Oral Biological and Medical Sciences, University of British Columbia, Vancouver, BC Canada; 2 UBC Centre for Blood Research, Life Sciences Institute, University of British Columbia, 2350 Health Sciences Mall, 4th Floor, Vancouver, BC V6T 1Z3 Canada; 3 Department of Medical Genetics, University of British Columbia, Vancouver, BC Canada; 4 Biomedical Research Centre, University of British Columbia, Vancouver, BC Canada; 5 Department of Pathology and Laboratory Medicine, Perelman School of Medicine, University of Pennsylvania, Philadelphia, PA USA; 6 Division of Hematology, The Children’s Hospital of Philadelphia, Philadelphia, PA USA; 7 Institute of Medical Biology, Polish Academy of Sciences, Lodz, Poland; 8 Department of Biochemistry and Molecular Biology, University of British Columbia, Vancouver, BC Canada

**Keywords:** Cell signalling, Periodontitis

## Abstract

Periodontitis is a chronic inflammatory disease characterized by the release of matrix metalloproteinases (MMPs) from resident connective tissue cells in tooth-supporting tissues (periodontium). Platelet activation, and the attendant release of pro-inflammatory chemokines such as platelet factor 4 (CXCL4/PF4), are associated with periodontitis although the associated biochemical pathways remain undefined. Here we report that recombinant PF4 is internalized by cultured human gingival fibroblasts (hGFs), resulting in significant (*p* < 0.05) upregulation in both the production and release of MMP-2 (gelatinase A). This finding was corroborated by elevated circulating levels of MMP-2 (*p* < 0.05) in PF4-overexpressing transgenic mice, relative to controls. We also determined that PF4 induces the phosphorylation of NF-κB; notably, the suppression of NF-κB signaling by the inhibitor BAY 11-7082 abrogated PF4-induced MMP-2 upregulation. Moreover, the inhibition of surface glycosaminoglycans (GAGs) blocked both PF4 binding and NF-κB phosphorylation. Partial blockade of PF4 binding to the cells was achieved by treatment with either chondroitinase ABC or heparinase III, suggesting that both chondroitin sulfate and heparan sulfate mediate PF4 signaling. These results identify a novel pathway in which PF4 upregulates MMP-2 release from fibroblasts in an NF-κB- and GAG-dependent manner, and further our comprehension of the role of platelet signaling in periodontal tissue homeostasis.

## Introduction

Tissue degradation resulting from chronic inflammation is largely mediated by enzymes termed matrix metalloproteinases (MMPs) and is observed in multiple human diseases, including atherosclerosis^1^, arthritis^2^, inflammatory bowel disease^3^ and chronic obstructive pulmonary disease^4^. Periodontitis is another chronic inflammatory disease initiated by periodontal pathogens in dental plaque^5^, most notably, the Gram-negative species *Porphyromonas gingivalis* and *Treponema denticola*^6^. The ensuing host immune response to these pathogens results in the progressive MMP-driven destruction of the periodontal tissues^7^. MMPs are normally synthesized and secreted as pro-enzymes that are activated upon removal of the autoinhibitory pro-domain^8^. MMP activity enables the physiologic remodeling of periodontal tissues; however, during the development of periodontitis, MMP activity is often dysregulated, leading to excessive breakdown of collagen, the major component of the connective tissue matrix^7^. Accordingly, multiple MMPs released by the inflammatory and resident connective tissue cells, are elevated in periodontal disease^7^. MMP expression and activity are regulated at various levels, including gene transcription, mRNA processing and degradation, and by activation and secretion^9^. MMPs are also regulated by micro RNAs (miRNA) and by tissue inhibitors of MMPs (TIMPs)^9^.

MMP-2, aka gelatinase A, is a key mediator of connective tissue degradation in both the gingival tissues^10^ and periodontal ligament^11^. Multiple lines of evidence support the role of MMP-2 in the pathogenesis of periodontitis. MMP-2 is reportedly activated by *P. gingivalis* in human gingival crevicular fluid^12^ and by *T. denticola* in human periodontal ligament fibroblasts^13^. Moreover, gingival MMP-2 expression is elevated in rats subjected to experimental periodontitis^14^, and in human gingival tissues harvested from patients with periodontitis^15^. Collectively, these data indicate that MMP-2 activity is correlated with disease activity, although little is known about the mechanisms that govern MMP-2 release from connective tissue cells. A better understanding of this area is of general relevance since MMP-2 has been identified as a potentially useful target for other diseases such as heart failure^16^ and neuroinflammatory disorders^17^.

The regulation of MMP expression in periodontal connective tissues is controlled by cytokines and chemokines from the immune cells recruited during inflammation; these cells include neutrophils^18^, macrophages^19^, and platelets^20^. While the role of platelets in hemostasis and thrombosis is well-documented, platelets also play a central role in the pathogenesis of multiple chronic inflammatory diseases^21^. This is likely due to the numerous pro-inflammatory cytokines and chemokines stored within the platelet granules^22^. Platelet factor 4 (PF4), aka CXCL4, is a 7.8-kDa chemokine that is a major constituent of platelet alpha-granules^23^. Upon activation, platelets release micromolar concentrations of PF4^24^. We previously reported elevated PF4 levels in the gingival crevicular fluid at periodontal disease sites, as well as in the plasma of patients with severe periodontitis^20^. Moreover, published data indicate that PF4 activates the nuclear factor kappa B (NF-κB) signaling pathway in endothelial cells^25^; importantly, NF-κB signaling has a documented role in the regulation of MMP expression^26,27^ and in the pathogenesis of periodontitis^28,29^.

Given the association between platelet activity and periodontitis^20,30,31^, and the lack of mechanistic knowledge of how platelet-derived cytokines influence disease pathogenesis, we hypothesized that PF4 promotes periodontal breakdown by upregulating MMP-2 production in the periodontal connective tissues. To test this hypothesis, we studied human gingival fibroblasts cultured in the presence of recombinant PF4. We discovered that PF4 increases NF-κB phosphorylation and promotes MMP-2 production and release from human gingival fibroblasts. We also determined that the PF4 signal is transduced through the glycosaminoglycans (GAG) on the cell surface. Moreover, circulating plasma levels of MMP-2 are elevated in transgenic mice that overexpress PF4, which further supports the notion that PF4 triggers MMP-2 release in connective tissues.

## Results

### PF4 enhances MMP-2 secretion by human gingival fibroblasts (hGFs)

**Figure 1:**
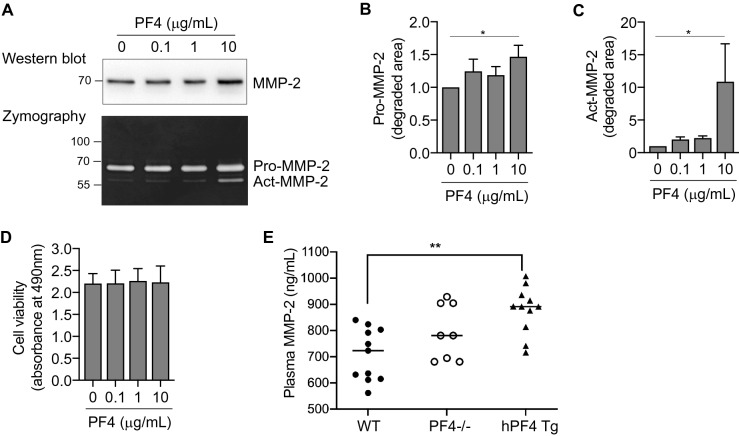
PF4 promotes the secretion of MMP-2. (**A**) Serum-starved human gingival fibroblasts (hGFs) were cultured for 24 h in the presence or absence of recombinant PF4 at the indicated concentrations. Cell culture supernatants were collected, resolved by SDS-PAGE, probed for MMP-2 (immunoblot, top panel), and also analyzed by gelatin zymography (bottom panel). (**B**)-(**C**)**.** Bar graphs depict gelatin degradation by pro-MMP-2 (**B**) and active MMP-2 (**C**), as quantified using ImageJ software. The gelatin-degraded area of the untreated samples (0) was set at 1. Data are mean ± SD and represent three independent experiments. *, *p* < 0.05, based on one-way ANOVA and Tukey’s multiple post-hoc comparison tests. (**D**) Bar graph confirming cell viability (as measured by the MTS assay) in the presence of recombinant PF4 at the indicated concentrations for 48 h. (**E**) Scatter plots represent levels of MMP-2 in plasma obtained from wild-type mice (*WT*, black circles), PF4-null mice (*PF4*^*-/-*^, open circles) and *WT* mice expressing the human PF4 transgene (*hPF4* Tg, black triangles). Data are mean ± SD and represent three independent experiments. **, *p* < 0.001 based on one-way ANOVA and Tukey's multiple comparisons test.

To improve our understanding of how platelet-derived chemokines drive pro-inflammatory signaling in the periodontal connective tissues, we used cultured human gingival fibroblasts and recombinant CXCL4 (PF4). We first measured MMP-2 in the cell culture supernatants by immunoblotting. Treatment with PF4 increased the amount of MMP-2 in the culture supernatants (Fig. 1A). We then used gelatin zymography to discern between pro-MMP-2 and active MMP-2; as expected, we observed two gelatinolytic bands, corresponding to pro-MMP-2 and active MMP-2, as previously reported^32^. Notably, we found that PF4 treatment increases, in a concentration-dependent manner, the gelatinolytic activity of both pro-MMP-2 and active MMP-2 (Fig. 1A). Relative to untreated controls, PF4 (10 μg/mL) increased gelatinolysis by pro-MMP-2 and active MMP-2 by 1.5-fold and tenfold, respectively (Fig. 1B,C). Since previous studies reported that PF4 stimulation protects monocytes from undergoing spontaneous apoptosis^33^, we wished to verify that the elevated MMP-2 secretion was not due to de-regulation of cell survival by PF4. Our data from the MTS assay confirm that PF4 upregulates MMP-2 secretion by hGFs without affecting cell viability (Fig. 1D). To evaluate this finding in an in vivo model, we measured circulating levels of MMP-2 in plasma obtained from wild-type (*WT*) mice, PF4-knockout (*Pf4*^*-/-*^) mice, and WT mice expressing the human *PF4* transgene (*hPF4* Tg). We noted that circulating MMP-2 levels were significantly (p < 0.05) higher in the *hPF4* Tg mice, which essentially overexpress PF4 (Fig. 1E). These data suggest that PF4-driven MMP-2 secretion from the connective tissues is reflected in the systemic circulation.

### PF4 increases intracellular MMP-2 protein production

**Figure 2:**
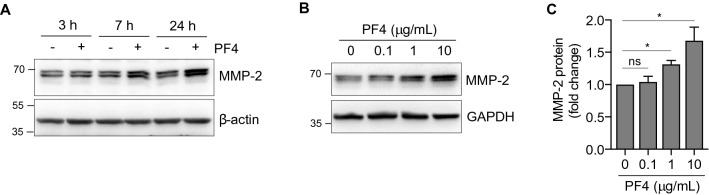
PF4 induces MMP-2 protein production. (**A**) Serum-starved hGFs were cultured in the absence (−) or presence (+) of recombinant PF4 (10 μg/mL) for the time periods indicated. Equal amounts of protein from cell lysates were resolved by SDS-PAGE and immunoblotted with antibodies against MMP-2 and beta-actin (β-actin). (**B**) Serum-starved cells were cultured in the absence (−) presence (+) of recombinant PF4 for 24 h at the concentrations indicated. Equal amounts of protein from cell lysates were resolved by SDS-PAGE and immunoblotted with an antibody against MMP-2. GAPDH is shown as a loading control. (**C**) Bar graph depicts the quantification of intracellular MMP-2 in PF4-treated cells, based on the immunoblots represented in (**B**). The ratio of MMP/GAPDH of the untreated sample (0) was set at 1. Data are mean ± SD and represent three independent experiments. *, *p* < 0.05, based on one-way ANOVA and Tukey’s post-hoc multiple comparison tests.

Having established that PF4 promotes MMP-2 secretion from hGFs, we then set out to determine whether this was the result of increased protein production. Immunoblots of whole cell lysates revealed that PF4 treatment increased intracellular MMP-2 in both a time-dependent (Fig. 2A) and a concentration-dependent manner (Fig. 2B). The increase in intracellular MMP-2 was statistically significant (*p* < 0.05) at PF4 doses of 1 μg/mL and 10 μg/mL (Fig. 2C).

### PF4 activates NF-κB signaling in gingival fibroblasts

**Figure 3:**
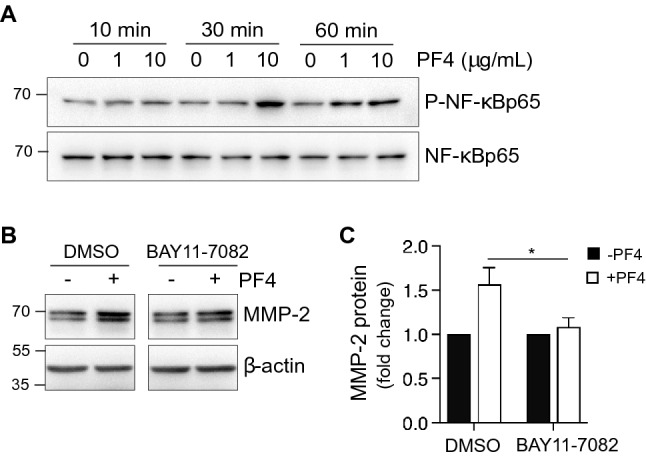
PF4 activates NF-κB signaling in hGFs. (**A**) Serum-starved cells were cultured in the absence or presence of recombinant PF4 at the concentrations and for the time periods indicated. Equal amounts of protein from cell lysates were resolved by SDS-PAGE and immunoblotted with antibodies against phospho-NF-kappaB (P-NF-κBp65). Total NF-κB is shown as a loading control. (**B**) Serum-starved cells were pre-treated with either DMSO vehicle or the NF-κB inhibitor BAY 11-7082 (5 µM) for 1 h, prior to treatment with recombinant PF4 (10 µg/mL) for 24 h. Cell lysates were resolved by SDS-PAGE; immunoblots were probed with antibody against MMP-2. β-actin is shown as a loading control. (**C**) Bar graph depicts intracellular MMP-2 in untreated (black bars) and PF4-treated cells (white bars) in the presence of vehicle (DMSO) or the NF-κB inhibitor BAY 11-7082, as represented in (**B**). Data are expressed relative to the untreated control groups (set at 1), and are mean ± SD from three independent experiments. *, p < 0.05, based on t-test.

To identify the signaling pathway through which PF4 upregulates MMP-2 translation and/or secretion, we focused on NF-κB, a signaling pathway reportedly activated by PF4^25^. Treatment of hGFs with 10 μg/mL of PF4 induced phosphorylation of NF-κB (Ser 536), with maximal phosphorylation at 30 min followed by a decline by 60 min post-treatment (Fig. 3A). We then evaluated the effect of NF-κB phosphorylation on PF4-induced MMP-2 expression, by pre-treating cells with the NF-κB pathway inhibitor BAY 11-7082, prior to the addition of PF4 (10 μg/mL). Blockade of NF-κB signaling with BAY 11-7082 (5 μM, 1 h) significantly (*p* < 0.05) attenuated PF4-driven increases in intracellular MMP-2 (Fig. 3B,C), suggesting that NF-κB phosphorylation is pivotal for PF4-driven increases in intracellular MMP-2. However, we did not detect any differences in MMP-2 mRNA between PF4-treated and untreated cells, based on real-time PCR (Supplemental Fig.). These results point to importance of NF-κB signaling in the context of PF4-mediated MMP-2 production but not MMP-2 gene transcription.

### PF4 is internalized and degraded by human gingival fibroblasts

**Figure 4:**
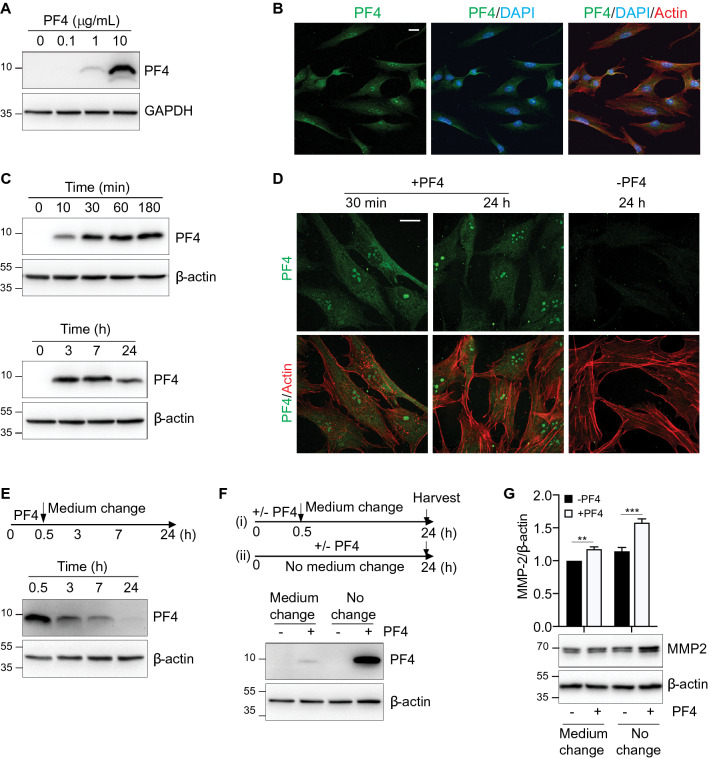
PF4 is internalized and degraded by hGFs. (**A**) Serum-starved cells were cultured in the absence or presence of recombinant PF4 at the indicated concentrations. Whole cell lysates were resolved by SDS-PAGE and the immunoblots were probed with an antibody against PF4. GAPDH is shown as a loading control. (**B**) Confocal micrographs illustrate the intracellular distribution of PF4 (green), DAPI (blue), and actin (red) in cells treated with recombinant PF4 (1 µg/mL) for 24 h prior to fixation. Cells were imaged with a Leica SP5 laser scanning confocal microscope. Bar = 20 µm. (**C**) Cells were treated with PF4 (10 µg/mL) for the indicated time periods. Cell lysates were resolved by SDS-PAGE and the immunoblots were probed with an antibody against PF4. β-actin is shown as a loading control. (**D**) Confocal micrographs illustrate the intracellular distribution of PF4 (green, top panels) and PF4/actin (green/red, bottom panels) in cells cultured in the absence (−) or presence (+) of recombinant PF4 for the indicated times prior to fixation and processing for microscopy. Slides were imaged with Zeiss spinning disk confocal microscope. Bar = 20 µm. (**E**) Serum-starved cells were cultured in the presence of recombinant PF4 (10 µg/mL) for 30 min (0.5 h) prior to replacement with PF4-free medium. Cells were harvested at the indicated time points. Cell lysates were resolved by SDS-PAGE and immunoblots probed with an anti-PF4 antibody. β-actin is shown as a loading control. (**F**) Top: Schematic illustration of the experimental strategy (medium change vs. no medium change): Scenario (i)—Medium change: cells were cultured in the absence (−) or presence (+) of recombinant PF4 for 30 min (0.5 h) prior to medium replacement with PF4-free medium. Scenario (ii)—No medium change: cells were cultured either in the absence (−) or presence (+) of recombinant PF4 for the duration of the 24 h time course. Cell culture supernatants were harvested, and cell lysates prepared, after 24 h. Bottom: Cell lysates were resolved by SDS-PAGE and immunoblots probed with anti-PF4 antibody. β-actin is shown as a loading control. (**G**) Top: Bar graph depicts the intracellular MMP-2 (expressed as the MMP-2:β-actin ratio) in cells cultured in the absence (-PF4, black bars) or presence (+ PF4, white bars) of recombinant PF4, both following withdrawal of PF4-containing medium (Medium change) or with the continued presence of PF4 (No change). The MMP-2:β-actin ratio of the untreated controls (-PF4, Medium change) was set at 1. Data are mean ± SD and represent three independent experiments. **, *p* < 0.01, ***, *p* < 0.001, based on two-way ANOVA and Tukey’s post-hoc multiple comparison tests. Bottom: Cell lysates were resolved by SDS-PAGE and immunoblots probed with anti-MMP-2 antibody. β-actin is shown as a loading control.

We then wished to gain insight into how PF4 is processed by hFGs. Western blotting of the lysates of hGFs incubated with PF4 indicate uptake of PF4 by hGFs in a dose-dependent manner (Fig. 4A). The internalization of PF4 was also verified by confocal microscopy; PF4 was distributed in the cytosol and the nuclei. Notably, PF4 existed in punctate structures in nuclei (Fig. 4B). To elucidate the time-course of PF4 uptake, cells were harvested at different times (10 min to 24 h) after PF4 treatment prior to cell lysis and immunoblotting (Fig. 4C). The uptake of PF4 occurred fairly rapidly, beginning as early as 10 min and peaking by 3 h post-treatment. Furthermore, we showed that the spatial distribution of PF4 at 30 min is similar to that at 24 h post-treatment (Fig. 4D). This result suggests that PF4 is rapidly internalized by hGFs and localized in various cellular compartments.

To investigate the stability of internalized PF4, cells were incubated with PF4-containing medium for 30 min and then replaced with PF4-free medium (Fig. 4E). The amount of cell-associated PF4 clearly decreased following media change, and was undetectable within 24 h (Fig. 4E), suggesting that PF4 is rapidly degraded intracellularly. To investigate if uptake of extracellular PF4 is continuous, cells were divided into two groups: (i) cells transiently treated with PF4 for 30 min and maintained in PF4-free medium for up to 24 h and (ii) cells maintained in PF4-containing medium for 24 h (Fig. 4F). The data indicate that intracellular PF4 remained abundant in the absence of media change but was virtually absent within 24 h of media change (Fig. 4F), suggesting that extracellular PF4 is continuously taken up and degraded by cells. Importantly, the persistence of extracellular PF4 was associated with a sustained production of intracellular MMP-2 (Fig. 4G). Collectively, these data indicate that extracellular PF4 is continuously taken up and degraded by hGFs, and further confirm the association between PF4 internalization and MMP-2 production.

### Cell surface glycosaminoglycans (GAGs) are required for intracellular uptake of PF4

**Figure 5:**
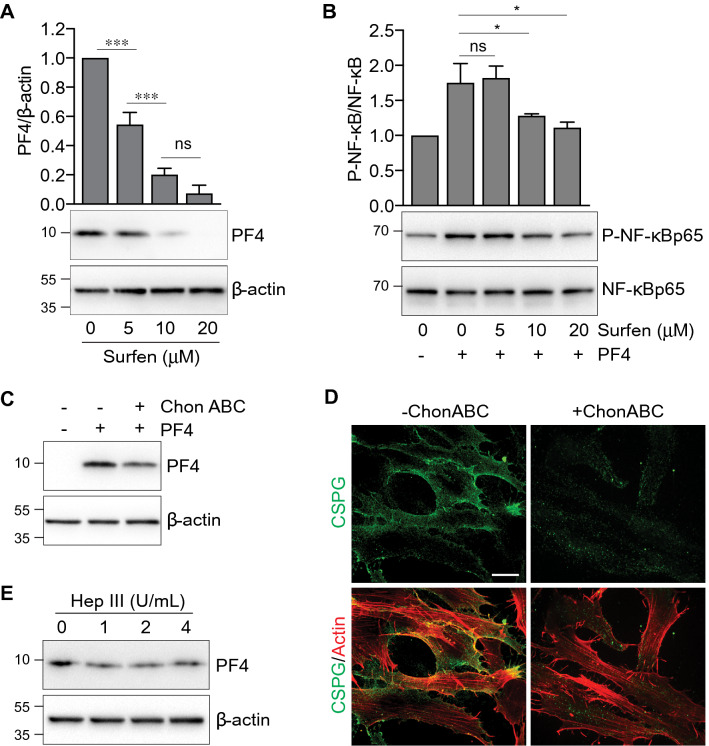
Removal of surface glycosaminoglycans (GAGs) blocks PF4 uptake and NF-κB signaling. (**A**) Serum-starved cells were incubated with various concentrations of the heparan sulfate antagonist Surfen at the indicated concentrations prior to treatment with recombinant PF4 (2 μg/mL) for 30 min. Whole cell lysates were resolved by SDS-PAGE and immunoblots were probed with an anti-PF4 antibody. β-actin is shown as a loading control. Top: bar graph depicts the intracellular PF4 uptake, expressed as the ratio of PF4:β-actin. Data are expressed as mean ± SD and represent three independent experiments. ***, p<0.001, based on ANOVA and Tukey's post-hoc multiple comparison tests. (**B**) Serum-starved cells were incubated with various concentrations of the heparan sulfate antagonist Surfen at the indicated concentrations prior to treatment with recombinant PF4 (2 μg/mL) for 30 min. Whole cell lysates were resolved by SDS-PAGE and immunoblots were probed with an antibody against phospho-NF-κB p65 (serine 536). Total NF-κB p65 is shown as a loading control. Top: bar graph depicts NF-κB phosphorylation, expressed as the ratio of phospho-NF-κB:NF-κB. Data are expressed as mean ± SD and represent three independent experiments. *, p<0.05, based on ANOVA and Tukey's post-hoc multiple comparison tests. (**C**) Cells were cultured in the absence (−) or presence (+) of chondroitinase ABC (Chon ABC, 0.5 U/mL) prior to treatment with PF4 (2 μg/mL) for 30 min. Whole cell lysates were resolved by SDS-PAGE and immunoblots were probed with an anti-PF4 antibody. β-actin is shown as a loading control. (**D**) Confocal micrographs illustrate surface chondroitin sulfate in cells cultured in the absence (−) or presence (+) of chondroitinase ABC, prior to fixation and immunostaining for chondroitin sulfate proteoglycan (CSPG, green) and actin (red). Bar = 20 μm. (**E**) Cells were cultured in the absence or presence of heparinase III at the indicated concentrations, prior to treatment with PF4 (2 μg/mL) for 30 min. Whole cell lysates were resolved by SDS-PAGE and immunoblots were probed with an anti-PF4 antibody. β-actin is shown as a loading control.

Several PF4 receptors have been identified, including CXCR3B, CXCR3A/B, and CCR1^34,35^ although these receptors are reportedly not expressed by hGFs^36^. Since PF4 binds cell surface glycosaminoglycans (GAGs) including chondroitin sulfate, dermatan sulfate, and heparan sulfate, we decided to evaluate the role of GAGs in PF4 uptake. We found that Bis-2-methyl-4-amino-quinolyl-6-carbamide (aka Surfen), a small molecule antagonist of cell surface GAGs^37^, inhibited PF4 uptake in a dose-dependent manner (Fig. 5A). PF4-induced phosphorylation of NF-κB was also attenuated by higher concentrations of Surfen (Fig. 5B). To further dissect out the relative importance of specific surface GAGs, we disrupted cell surface GAGs using 2 specific GAG lyases. First, pre-treatment of cells with chondroitinase ABC (1 h), which targets chondroitin sulfate and dermatan sulfate^38^, decreased cell-associated PF4 (Fig. 5C). The efficiency of chondroitin sulfate peptidoglycan (CSPG) degradation by chondroitinase ABC was confirmed by confocal microscopy (Fig. 5D). Second, since chondroitinase ABC partially blocked PF4 internalization, we then examined the role of heparan sulfate, another critical component of cell surface GAGs. Pretreatment of hGFs with heparinase III (1–4 U/mL) blocked intracellular uptake of PF4 (Fig. 5E). These data clearly indicate that PF4 internalization is contingent on sulfated GAGs, including chondroitin sulfate, dermatan sulfate, and heparan sulfate.

## Discussion

The regulation of MMP expression and secretion is a critical aspect of the pathogenesis of periodontitis^7^. Platelet activity is associated with periodontitis^20,30,31^ but the mechanisms underlying the role of platelets in periodontal disease pathogenesis is unclear. We therefore studied how PF4 (one of the most abundant platelet-derived chemokines) signals to the gingival fibroblast (the main connective tissue cell of the periodontium). We discovered that PF4 promotes the production and/or release of MMP-2 from gingival fibroblasts. We also identified that PF4 increases MMP-2 protein expression in a glycosaminoglycan (GAG)- and NF-κB-dependent manner.

### PF4 upregulates MMP-2 protein expression

MMP expression and activity are tightly regulated at multiple checkpoints including transcription, regulation of mRNA half-life, enzyme activation, and secretion^9^. Like most MMPs, MMP-2 is synthesized and secreted as a pro-enzyme that is subsequently activated^8^. We found that intracellular and secreted MMP-2 were elevated following exposure to PF4 in a dose-dependent manner (10 μg/mL or 1.28 μM) which is consistent with local PF4 concentrations measured following platelet activation^24^. Since increases were observed in both pro-MMP-2 and active MMP-2, it appears likely that PF4 also plays a role in MMP-2 activation. Our results are further corroborated by the elevated plasma levels of MMP-2 measured in PF4-overexpressing mice; this novel finding directly supports the notion that PF4 increases the synthesis and/or secretion of MMP-2 from connective tissue cells, which is ultimately reflected in the systemic circulation.

### PF4 activates NF-κB signaling in gingival fibroblasts

We evaluated NF-κB signaling in gingival fibroblasts, as PF4 reportedly activates NF-κB in endothelial cells^25^. Moreover, NF-κB signaling has a well-documented association with chronic inflammatory disease^39^; notably, inhibition of NF-κB signaling suppresses periodontitis in experimental murine models^28,29^. Our data revealed that PF4 increases the phosphorylation of NF-κB at Ser536, which is reportedly important for pro-inflammatory NF-κB signaling^40^. Our data also indicate that blockade of NF-κB phosphorylation with the BAY 11-7082 inhibitor reduced MMP-2 production. While NF-κB phosphorylation at Ser536 reportedly enhances transcription activity^41^, we did not detect an increase in MMP-2 mRNA levels following PF4 treatment. We therefore surmise that the translation, and not transcription, of MMP-2 mRNA could be upregulated following PF4-induced phosphorylation of NF-κB. This notion is partially supported by the documented interactions between NF-κB and the translation factor eukaryotic initiation factor-2 (eIF2)^42,43^. In any case, our data are consistent with a pro-inflammatory role for PF4 and NF-κB signaling in the periodontium.

### PF4 targets cell surface GAGs

The splice variant of the CXCR3 receptor, termed CXCR3B, was identified as a PF4 receptor in microvascular endothelial cells^34^. A recent report also identified CCR1 as a PF4 receptor that controls monocyte migration^35^ although neither CCR1 nor CXCR3 were detected on the surface of human gingival fibroblasts^36^. We therefore focused on glycosaminoglycans (GAGs) which are also established PF4 ligands^44–46^. Previous studies indicate that chondroitin sulfate mediates PF4 binding to platelets^46^, while heparan sulfate mediates PF4 binding to endothelial cells^44^. However, in our study, we found that both chondroitin sulfate and heparan sulfate mediate PF4 binding to, and internalization by, gingival fibroblasts. Importantly, both PF4 internalization and NF-κB phosphorylation were attenuated after neutralization of the surface GAGs, which underscores the importance of PF4 internalization for its biological activity.

One limitation of our study is that it does not precisely identify how PF4-driven NF-κB signaling results in increased MMP-2 production and/or secretion. Given the complexity of pre- and post-transcriptional regulation of MMPs^9^, and the involvement of multiple intermediates in the NF-κB pathway^39^, it would be interesting to determine which downstream targets of NF-κB could be responsible for increasing protein production or exocytosis in gingival fibroblasts. Nonetheless, our novel findings shed light on how platelet activation contributes to tissue degradation in the periodontium, thus providing a partial explanation for the previously reported associations between platelet activity and periodontitis^20,30^. In conclusion, we report that PF4 binds to gingival fibroblasts via GAGs, and its subsequent internalization activates the NF-κB signaling pathway and increases MMP-2 protein synthesis and release. Future studies could identify the specific proteins activated by PF4 in the NF-κB pathway, as well as the mechanism(s) by which they upregulate MMP-2 production.

## Methods

### Reagents

Recombinant PF4, chondroitinase ABC, gelatin and Surfen were purchased from Millipore-Sigma (Oakville, ON, Canada). Antibodies against MMP-2, PF4, and chondroitin sulfate, as well as the NF-κB inhibitor BAY 11-7082, were obtained from Abcam (Cambridge, MA). The anti-GAPDH antibody was from HyTest (Turku, Finland). The anti-β-actin antibody was from Santa Cruz Biotechnologies (Dallas, TX). Heparinase III, and antibodies against phospho-NF-κB (Ser536), total NF-κB, and horseradish peroxidase-conjugated secondary antibodies were from Cell Signaling Technologies (Billerica, MA). The Alexa-Fluor-488-conjugated secondary antibody, and Alexa-Fluor-568 phalloidin were from Life Technologies (Grand Island, NY).

### Cell culture

All cell culture reagents were obtained from Gibco (ThermoFisher Scientific, Grand Island, NY). hTERT immortalized human gingival fibroblasts (ABM Good, Richmond, BC, Canada) were cultured in DMEM supplemented with 10% FBS and antibiotics (100 U/mL penicillin and 100 µg/mL streptomycin). Cells (passages 4–10) were seeded in 6-well culture plates at a density of 3 × 10^5^ cells/well. Cells were serum-starved for 24 h prior to PF4 treatment.

### Immunoblotting

Cells were lysed in RIPA buffer (20 mM Tris–HCl, 100 mM NaCl, 1% (v/v) Triton X-100, 0.1% (w/v) SDS, 0.5% (w/v) sodium deoxycholate, 5 mM EDTA, pH = 7.6) supplemented with protease and phosphatase inhibitors. Cell lysates were clarified by centrifugation at 16,000* g* for 15 min at 4 °C. 10–20 µg of protein was resolved by SDS-PAGE, transferred onto PVDF membranes, followed by incubation with a blocking buffer (1 h) and primary antibodies (4 °C overnight). Proteins were detected with an HRP-conjugated secondary antibody and enhanced chemiluminescence. Densitometric quantification was performed using Image Lab software.

### Mice

Pf4-null mice (*Pf4*^-/-^ aka mPF4KO) and human PF4 transgenic (*hPF4*-Tg) mice^47^ were backcrossed to C57Bl/6 J (JAX#:000664) for at least 6 generations and maintained at the University of British Columbia (UBC) Biomedical Research Centre specific-pathogen free (SPF) facility. All mice were treated according to guidelines from the Canadian Council on Animal Care (CCAC) and as approved by the UBC Animal Care Committee. Whole blood from wild-type (*WT*), *Pf4*^-/-^, and *hPF4*-Tg mice was collected by retro-orbital plexus bleeding in an anticoagulant buffer containing 3.2% sodium citrate. Platelet-poor plasma was obtained by sequential centrifugation of whole blood and the resultant platelet-rich plasma (PRP). The platelet-poor plasma was stored at − 80 °C until use; plasma MMP-2 levels were measured with an enzyme-linked immunosorbent assay (ELISA) kit (Abcam, Cambridge, MA) in accordance with the manufacturer’s instructions.

### Gelatin zymography

Cell culture supernatants were clarified by centrifugation at 3000* g* for 10 min at 4 °C to remove cellular debris. The supernatants were stored in aliquots at − 80 °C until use. The samples, under non-reducing conditions, were run on a 10% SDS-PAGE gel containing 0.1% gelatin. Gels were incubated with 2.5% (v/v) Triton X-100 for 30 min at room temperature to remove SDS, rinsed in water, and incubated in a buffer (50 mM Tris–HCl pH 8, 150 mM NaCl, 5 mM CaCl_2_) for 18 h at 37 °C. Gels were then stained with 0.5% Coomassie Blue, destained, and imaged using a digital scanner. The area of the gelatinase bands was quantified using ImageJ software (National Institutes of Health, Bethesda, MD).

### Gene expression analysis

RNA was extracted using an RNeasy mini kit (Qiagen, Hilden, Germany). One microgram of RNA was reverse-transcribed using qScript cDNA SuperMix (QuantaBiosciences, Gaithersburg, MD) according to the manufacturer’s protocol. Real-time qPCR was performed using SsoFast™ EvaGreen Supermix and CFX Connect Real-time PCR Detection System (Bio-Rad, Hercules, CA). qPCR (∆∆CT) was analyzed using forward and reverse primer sets for *MMP2* (F: 5′-*AGGGCACATCCTATGACAGC*-3′, R: 5′-*ATTTGTTGCCCAGGAAAGTG*-3′), and *GAPDH* (F: 5′- *AGGGCTGCTTTTAACTCTGGT*-3′, R: 5′-*CCCCACTTGATTTTGGAGGGA*-3′)^48^. 25 ng of cDNA template was used for each reaction. The qPCR conditions were set at 98 °C for 2 min followed by 40 cycles of 98 °C for 5 s and 60 °C for 5 s. The threshold cycle values were used to calculate relative RNA expression levels. Values were normalized to endogenous *GAPDH* transcripts.

### Immunocytochemistry

Cells on 8-well chamber slides were fixed with 4% paraformaldehyde for 10 min. To detect PF4, cells were permeabilized with 0.1% (v/v) Triton X-100 for 5 min, blocked with 5% goat serum for 1 h, incubated with the appropriate primary antibody overnight at 4 °C, followed by the appropriate secondary antibody. To detect cell surface chondroitin sulfate, cells were not permeabilized prior to incubation with the anti-chondroitin sulfate antibody. Slides were imaged with a Zeiss spinning disk confocal microscope and SlideBook software or a Leica SP5 laser scanning confocal microscope and LAS-AF software.

### Cell viability assay

The viability of serum-starved hGFs was evaluated using the CellTiter 96 AQ_ueous_ One Solution Reagent (Promega, Madison, WI). Briefly, 20 μl of CellTiter 96 AQ_ueous_ One Solution Reagent was added into each well of a 96-well plate containing 100 μl of serum-free media. The MTS tetrazolium compound in the reagent solution was bioreduced by cells into a colored formazan product; the absorbance was recorded at 490 nm.

### Statistical analysis

Quantitative data from the zymography, immunoblots , and cell viability assays are presented as mean ± standard deviation (SD). Statistical analysis was performed with GraphPad Prism software (La Jolla, CA, USA) using an analysis of variance (ANOVA) with Tukey’s post-hoc multiple comparisons tests (when comparing 3 or more groups)or the Student's t-test (when comparing 2 groups). Statistical significance was set at *p* < 0.05.

The portion of the study involving mice adhered to the Animal Research: Reporting In Vivo Experiments (ARRIVE) guidelines (2.0).

## Supplementary Information

Supplementary Information.

## Data Availability

The data that support the conclusions of this study are included in the article. The original images for all of the immunoblots presented in the Figures are provided as Supplementary Information. Additional information is available from the corresponding author on reasonable request.

## References

[1] Wang X, Khalil RA (2018). Matrix metalloproteinases, vascular remodeling, and vascular disease. Adv. Pharmacol..

[2] Burrage PS, Mix KS, Brinckerhoff CE (2006). Matrix metalloproteinases: Role in arthritis. Front. Biosci..

[3] de Bruyn M (2016). The molecular biology of matrix metalloproteinases and tissue inhibitors of metalloproteinases in inflammatory bowel diseases. Crit. Rev. Biochem. Mol. Biol..

[4] Navratilova Z, Kolek V, Petrek M (2016). Matrix metalloproteinases and their inhibitors in chronic obstructive pulmonary disease. Arch. Immunol. Ther. Exp. (Warsz).

[5] Pihlstrom BL, Michalowicz BS, Johnson NW (2005). Periodontal diseases. Lancet.

[6] Holt SC, Ebersole JL (2005). Porphyromonas gingivalis, Treponema denticola, and Tannerella forsythia: The "red complex", a prototype polybacterial pathogenic consortium in periodontitis. Periodontol.

[7] Sorsa T (2006). Matrix metalloproteinases: Contribution to pathogenesis, diagnosis and treatment of periodontal inflammation. Ann. Med..

[8] Nagase H, Visse R, Murphy G (2006). Structure and function of matrix metalloproteinases and TIMPs. Cardiovasc. Res..

[9] Hadler-Olsen E, Fadnes B, Sylte I, Uhlin-Hansen L, Winberg JO (2011). Regulation of matrix metalloproteinase activity in health and disease. FEBS J..

[10] Creemers LB (1998). Gelatinase A (MMP-2) and cysteine proteinases are essential for the degradation of collagen in soft connective tissue. Matrix Biol..

[11] Kawagoe M, Tsuruga E, Oka K, Sawa Y, Ishikawa H (2013). Matrix metalloproteinase-2 degrades fibrillin-1 and fibrillin-2 of oxytalan fibers in the human eye and periodontal ligaments in vitro. Acta Histochem. Cytochem..

[12] Grayson R, Douglas CW, Heath J, Rawlinson A, Evans GS (2003). Activation of human matrix metalloproteinase 2 by gingival crevicular fluid and Porphyromonas gingivalis. J. Clin. Periodontol..

[13] Malone ET (2021). Treponema denticola-Induced RASA4 upregulation mediates cytoskeletal dysfunction and MMP-2 activity in periodontal fibroblasts. Front. Cell Infect. Microbiol..

[14] Silva JA (2008). The influence of type I diabetes mellitus on the expression and activity of gelatinases (matrix metalloproteinases-2 and -9) in induced periodontal disease. J. Periodontal Res..

[15] Ateia IM (2018). Treponema denticola increases MMP-2 expression and activation in the periodontium via reversible DNA and histone modifications. Cell Microbiol..

[16] Goncalves PR, Nascimento LD, Gerlach RF, Rodrigues KE, Prado AF (2022). Matrix metalloproteinase 2 as a pharmacological target in heart failure. Pharmaceuticals (Basel).

[17] Hannocks MJ (2019). The gelatinases, MMP-2 and MMP-9, as fine tuners of neuroinflammatory processes. Matrix Biol..

[18] Vitkov L (2021). Neutrophils orchestrate the periodontal pocket. Front. Immunol..

[19] Wang W, Zheng C, Yang J, Li B (2021). Intersection between macrophages and periodontal pathogens in periodontitis. J. Leukoc. Biol..

[20] Brousseau-Nault M, Kizhakkedathu JN, Kim H (2017). Chronic periodontitis is associated with platelet factor 4 (PF4) secretion: A pilot study. J. Clin. Periodontol..

[21] Cognasse F (2022). Platelets as key factors in inflammation: Focus on CD40L/CD40. Front. Immunol..

[22] Karshovska E, Weber C, von Hundelshausen P (2013). Platelet chemokines in health and disease. Thromb. Haemost..

[23] Lambert MP (2007). Platelet factor 4 is a negative autocrine in vivo regulator of megakaryopoiesis: Clinical and therapeutic implications. Blood.

[24] Brandt E (2000). The beta-thromboglobulins and platelet factor 4: Blood platelet-derived CXC chemokines with divergent roles in early neutrophil regulation. J. Leukoc. Biol..

[25] Yu G, Rux AH, Ma P, Bdeir K, Sachais BS (2005). Endothelial expression of E-selectin is induced by the platelet-specific chemokine platelet factor 4 through LRP in an NF-kappaB-dependent manner. Blood.

[26] Hou SM, Hou CH, Liu JF (2017). CX3CL1 promotes MMP-3 production via the CX3CR1, c-Raf, MEK, ERK, and NF-kappaB signaling pathway in osteoarthritis synovial fibroblasts. Arthritis. Res. Ther..

[27] Zhang Z (2022). IQGAP1 enhances cell invasion and matrix metalloproteinase-2 expression through upregulating NF-kappaB activity in esophageal squamous cell carcinoma cells. Gene.

[28] Wang B (2021). TPCA-1 negatively regulates inflammation mediated by NF-kappaB pathway in mouse chronic periodontitis model. Mol. Oral Microbiol..

[29] Wang J (2021). Inhibition of nuclear factor kappa B inducing kinase suppresses inflammatory responses and the symptoms of chronic periodontitis in a mouse model. Int. J. Biochem. Cell Biol..

[30] Papapanagiotou D (2009). Periodontitis is associated with platelet activation. Atherosclerosis.

[31] Zhan Y (2020). Platelets as inflammatory mediators in a murine model of periodontitis. J. Clin. Periodontol..

[32] Toth M, Fridman R (2001). Assessment of gelatinases (MMP-2 and MMP-9 by gelatin zymography. Methods Mol. Med..

[33] Scheuerer B (2000). The CXC-chemokine platelet factor 4 promotes monocyte survival and induces monocyte differentiation into macrophages. Blood.

[34] Lasagni L (2003). An alternatively spliced variant of CXCR3 mediates the inhibition of endothelial cell growth induced by IP-10, Mig, and I-TAC, and acts as functional receptor for platelet factor 4. J. Exp. Med..

[35] Fox JM (2018). CXCL4/Platelet Factor 4 is an agonist of CCR1 and drives human monocyte migration. Sci. Rep..

[36] Buskermolen JK, Roffel S, Gibbs S (2017). Stimulation of oral fibroblast chemokine receptors identifies CCR3 and CCR4 as potential wound healing targets. J. Cell. Physiol..

[37] Schuksz M (2008). Surfen, a small molecule antagonist of heparan sulfate. Proc. Natl. Acad. Sci. U.S.A..

[38] Shaya D (2008). Characterization of chondroitin sulfate lyase ABC from Bacteroides thetaiotaomicron WAL2926. Biochemistry.

[39] Barnabei L, Laplantine E, Mbongo W, Rieux-Laucat F, Weil R (2021). NF-kappaB: At the borders of autoimmunity and inflammation. Front. Immunol..

[40] Buss H (2012). Cyclin-dependent kinase 6 phosphorylates NF-kappaB P65 at serine 536 and contributes to the regulation of inflammatory gene expression. PLoS ONE.

[41] Chen LF (2005). NF-kappaB RelA phosphorylation regulates RelA acetylation. Mol. Cell. Biol..

[42] Deng J (2004). Translational repression mediates activation of nuclear factor kappa B by phosphorylated translation initiation factor 2. Mol. Cell. Biol..

[43] Jiang HY, Wek RC (2005). GCN2 phosphorylation of eIF2alpha activates NF-kappaB in response to UV irradiation. Biochem. J..

[44] Luster AD, Greenberg SM, Leder P (1995). The IP-10 chemokine binds to a specific cell surface heparan sulfate site shared with platelet factor 4 and inhibits endothelial cell proliferation. J. Exp. Med..

[45] Petersen F, Brandt E, Lindahl U, Spillmann D (1999). Characterization of a neutrophil cell surface glycosaminoglycan that mediates binding of platelet factor 4. J. Biol. Chem..

[46] Rauova L (2006). Role of platelet surface PF4 antigenic complexes in heparin-induced thrombocytopenia pathogenesis: Diagnostic and therapeutic implications. Blood.

[47] Eslin DE (2004). Transgenic mice studies demonstrate a role for platelet factor 4 in thrombosis: Dissociation between anticoagulant and antithrombotic effect of heparin. Blood.

[48] Herath TD (2013). The expression and regulation of matrix metalloproteinase-3 is critically modulated by *Porphyromonas gingivalis* lipopolysaccharide with heterogeneous lipid A structures in human gingival fibroblasts. BMC Microbiol..

