# Supplementary material for: Platelet factor 4 (CXCL4/PF4) upregulates matrix metalloproteinase-2 (MMP-2) in gingival fibroblasts
Source: Sci Rep. 2022 Nov 3;12:18636. doi: 10.1038/s41598-022-19850-w (PMC9633774; doi:10.1038/s41598-022-19850-w)

SUPPLEMENTAL MATERIAL:

Platelet factor 4 (CXCL4/PF4) upregulates matrix metalloproteinase-2 (MMP-2) in gingival fibroblasts

Hoa T. Le, Kalyan Golla, Ryan Karimi, Michael R. Hughes, Flavia Lakschevitz, Douglas B. Cines, M. Anna Kowalska, Mortimer Poncz, Kelly M. McNagny, Lari Häkkinen and Hugh Kim

SUPPLEMENTAL FIGURE LEGEND

Supplemental Figure. PF4 does not upregulate MMP-2 gene transcription. A-B. Bar graphs depict MMP-2 gene transcription in cells cultured in the absence (-) or presence (+) of recombinant PF4 at the indicated concentrations for 6 h (**A**) and 24 h **(B)**. Gene expression was normalized to the reference gene *GAPDH*. The untreated sample (0) was set at 1. Data are expressed as mean ± SD and represent three independent experiments.

Supplemental Figure

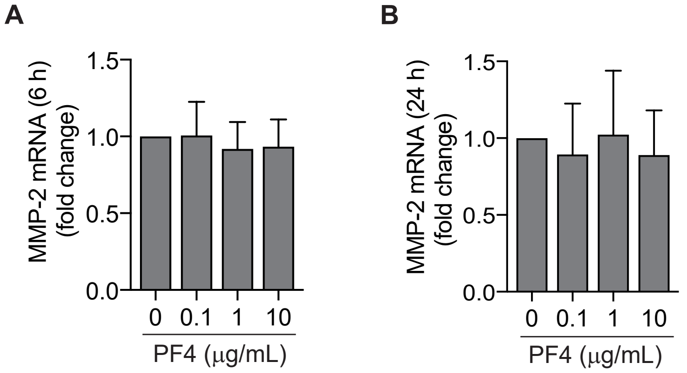

Supplement: Supplementary file 1 — Supplementary Information. [file 41598_2022_19850_MOESM1_ESM.pdf]
